# A replica exchange umbrella sampling (REUS) approach to predict host–guest binding free energies in SAMPL8 challenge

**DOI:** 10.1007/s10822-021-00385-7

**Published:** 2021-05-03

**Authors:** Mahdi Ghorbani, Phillip S. Hudson, Michael R. Jones, Félix Aviat, Rubén Meana-Pañeda, Jeffery B. Klauda, Bernard R. Brooks

**Affiliations:** 1 Laboratory of Computational Biology, National Heart, Lung and Blood Institute, National Institutes of Health, Bethesda, MD 20892 USA; 2 Department of Chemical and Biomolecular Engineering, University of Maryland, College Park, 20740 USA

**Keywords:** Binding free energy, Replica exchange umbrella sampling, Molecular dynamics, Weighted histogram analysis

## Abstract

In this study, we report binding free energy calculations of various drugs-of-abuse to Cucurbit-[8]-uril as part of the SAMPL8 blind challenge. Force-field parameters were obtained from force-matching with different quantum mechanical levels of theory. The Replica Exchange Umbrella Sampling (REUS) approach was used with a cylindrical restraint to enhance the sampling of host–guest binding. Binding free energy was calculated by pulling the guest molecule from one side of the symmetric and cylindrical host, then into and through the host, and out the other side (bidirectional) as compared to pulling only to the bound pose inside the cylindrical host (unidirectional). The initial results with force-matched MP2 parameter set led to RMSE of 4.68 ${\text{kcal}}/{\text{mol}}$ from experimental values. However, the follow-up study with CHARMM generalized force field parameters and force-matched PM6-D3H4 parameters resulted in RMSEs from experiment of $2.65$ and $1.72 {\text{kcal}}/{\text{mol}}$, respectively, which demonstrates the potential of REUS for accurate binding free energy calculation given a more suitable description of energetics. Moreover, we compared the free energies for the so called bidirectional and unidirectional free energy approach and found that the binding free energies were highly similar. However, one issue in the bidirectional approach is the asymmetry of profile on the two sides of the host. This is mainly due to the insufficient sampling for these larger systems and can be avoided by longer sampling simulations. Overall REUS shows great promise for binding free energy calculations.

**Supplementary Information:**

The online version contains supplementary material available at 10.1007/s10822-021-00385-7.

## Introduction

Binding free energy prediction between a receptor and a ligand is a crucial task for small-molecule drug discovery, and computational techniques have recently found great utility for this pivotal task [1–3]. Among these, molecular dynamics simulations are frequently used for virtual screening and lead optimization of drug candidates. A major goal of computer-aided drug design is to decrease the time and cost of lead optimization. This requires a detailed understanding of the intrinsic protein–ligand interactions, which are mainly studied via free energy calculation methods.

Due to the complexity in protein–ligand complexes, smaller representative systems are often used to assess the prediction accuracy of free energy methods, avoiding the sizeable conformational space that needs to be sampled for protein–ligand systems [3–5]. Statistical Assessment of the Modeling of Proteins and Ligands (SAMPL) blind challenges have provided a unique opportunity to validate different computational methods for quantitative prediction of binding free energies.

**Fig. 1:**
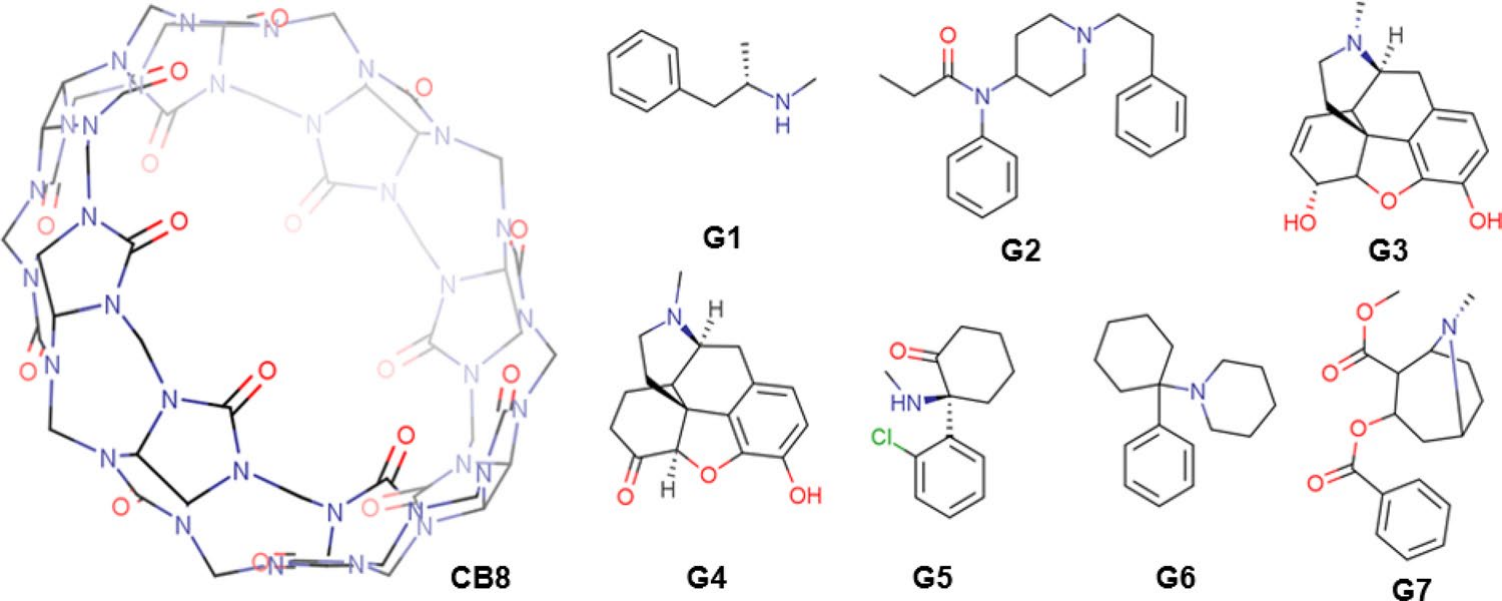
Host and guest systems for SAMPL8 challenge

Recent SAMPL challenges have utilized methylene-bridged glycoluril oligomer (CB[n] family) as a receptor with different guests of interest. The CB[n] family of macromolecules has a multitude of potential applications, such as drug delivery vehicles, molecular switches and catalysts [4–6]. For the SAMPL8 challenge, the host molecule was Cucurbit-[8]uril (CB[8]) (Fig. 1), a methylene-bridged macrocycle with eight glycoluril units. CB[8] has a hydrophobic core and a ring of carbonyl groups on both the top and bottom of its cylindrical shape and has attracted considerable interest due to its carbonyl portal and symmetrical structure. The SAMPL8 challenge featuring “drugs of abuse” presented 7 guest molecules including Methamphetamine (G1), Fentanyl (G2), morphine (G3), Hydromorphone (G4), Ketamine (G5), Phencyclidine (G6) and Cocaine (G7) shown in Fig. 1. Experimental ITC binding affinities for all host–guest systems were provided by the organizers after the challenge [7].

The quality of a binding free energy calculation hinges on how well the two following aspects are considered: (1) accuracy of the energetic description for the system of interest (e.g., the force-field employed) and (2) the method utilized for computing the free energy. Historically, Umbrella Sampling (US) can be used to calculate binding free energy across a physically relevant reaction coordinate [8, 9]. In this method, a harmonic restraint is placed at successive points along the reaction coordinate. The weighted histogram analysis method (WHAM) is used to convert the biased probabilities obtained from US into potentials of mean force (PMF) along the reaction coordinate [10, 11]. US simulations require thorough equilibrium sampling of all relevant conformational/configurational degrees of freedom, which can be computationally expensive.

A major problem with US is sampling insufficiency. For instance, in the bound pose, the ligand cannot sample different conformations due to the geometric restraints of the bound pose. However, generalized-ensemble methods can overcome this sampling inefficiency. In this case, states are weighted by a probability weight factor to allow for a random walk in potential energy space [12]. This allows the simulation to escape from any local energetic barrier and sample a broad configurational space. One such methods is Replica Exchange Umbrella Sampling (REUS) where exchange attempts are made between neighboring umbrella potentials. This method allows for identifying multiple binding poses with high resolution, that is, with respect to the limitations of the chosen force-field [13–16].

In the first step, Paramchem was used to generate CHARMM parameters for the host and guest molecules [17, 18]. Another possible approach is the Force Matching method, where one choses the MM energetic parameters that best reproduces the forces at a higher-level of theory (QM) [19–26]. In this approach, the parameter transferability is sacrificed for the sake of producing a highly specific forcefield description [22, 27]. Force-matching was successfully used in previous SAMPL competitions to generate parameters for the host–guest systems. Hudson and co-workers followed this approach during the SAMPL6 challenge and computed the free energies with a Double Decoupling method, and a similar procedure was utilized in this work employing the ForceSolve software [28].

This paper is organized as follows: in the Methods section, we describe the force-field parameterization, force matched parameter generation, simulation parameters, REUS setup and corrections to free energy. In the Results and Discussion section, we provide the computed free energies with various parameter sets. Then we describe some of the subtleties in the free energy calculation using REUS method.

## Methods

### Parameterization

Force field parameters for host and guests employ the following CHARMM potential energy function [29–32]. The potential energy function for nonbonded and bonded energies is given by:

1 $$U_{nonbonded} = \mathop \sum \limits_{nonbonded} \frac{{q_{i} q_{j} }}{{4\pi_{0} r_{ij} }} +_{ij} \left[ {\left( {\frac{{R_{min,ij} }}{{r_{ij} }}} \right)^{12} - 2\left( {\frac{{R_{min,ij} }}{{r_{ij} }}} \right)^{6} } \right]$$

2 $$U_{bonded} = \mathop \sum \limits_{bond} K_{b} \left( {b - b_{0} } \right)^{2} + \mathop \sum \limits_{angle} K_{\theta } \left( {\theta - \theta_{0} } \right)^{2} + \mathop \sum \limits_{dihedral} K_{\chi } \left( {1 + \cos \left( {n\chi - \delta } \right)} \right) + \mathop \sum \limits_{improper} K_{imp} \left( {\phi - \phi_{0} } \right)^{2}$$

In Equation (1) the first term in nonbonded potential energy is the Coulombic electrostatic interactions between atoms, where and $q_{j}$ are partial charges of atoms $i$ and $j$, $r_{ij}$ is the distance between the two atoms and $\varepsilon_{0}$ is the vacuum permittivity. The second term in the expression for nonbonded energy is the van der Waals (vdW) interaction by standard 6–12 Lennard–Jones (LJ) potential where $R_{min,ij}$ is the minimum distance and $\varepsilon_{ij}$ is the depth of the minimum in LJ function. To obtain the LJ parameters, Lorentz-Berthelot combination rule are used, where $\varepsilon_{ij}$ values are based on geometric mean of $\varepsilon_{i}$ and $\varepsilon_{j}$ whereas $R_{min,ij}$ is based on the arithmetic mean of $R_{min,i}$ and $R_{min,j}$. The intermolecular (bonded) portion of potential energy function in Eq. (2) includes terms for bond, bond angle, dihedral angle and improper torsion angle terms. Details of the potential energy function and specific terms in the equation can be found elsewhere [17, 18, 32].

Parameterization for host and guests was performed with a similar approach to our previous work on SAMPL6 challenge [33]. Specifically, bonded and dihedral parameters were determined using intramolecular force matching atomic charges were assigned via QM charge fitting, and LJ parameters were carried over from CGenFF [17, 18]. As the host was identical to the host in SAMPL6 challenge, and based on the good performance of those parameters, the SAMPL6 host parameters were reused (the so-called S6 parameters) in a few of our calculations.

Assignment of partial charges was performed through a combination of QM geometry optimization and single-point calculations with an implicit solvent model. Gas-phase geometry optimizations were performed for the guests at the MP2/6-31G(d) level of theory, whereas the host was optimized with B3LYP/6-31G(d) to ease computational expense. Following this, partial charges were then obtained via CM5 symmetrized charge fitting on the geometry optimized structures using HF/6-311G(d,p) with PCM implicit solvent. This particular combination (e.g. HF/6-311G(d,p) with PCM and CM5 symmetrized charges) was selected based on the best result from an internal benchmark comparing the performance of various QM charge fitting schemes on a collection of small molecules against charges found in CGenFF. Following determination of partial charges, LJ parameters were obtained from CGenFF through the ParamChem server [17, 18, 34].

Using the initial CGenFF guest parameters obtained with ParamChem (S6 parameters were initialized for the host), 100 ns of gas-phase Langevin Dynamics (LD) was performed via CHARMM with a coordinate snapshot saving frequency of 10 ${\text{ps}}$, a temperature of 300 K, collision frequency of $5 {\text{ps}}^{ - 1}$, a timestep of $1 {\text{fs}}$ and not tapering on non-bonded energetics. Each configuration collected during the LD simulations (10,000 per molecule) underwent force calculations at the GFN-2, PM6-D3H4, MP2/6-31G(d) (B3LYP/6-31G(d) for the host), and ${\upomega }$ B97XD/def2-SVP levels of theory using XTB [35], MOPAC, and PSI4 respectively [36, 37]. Classical van-der-Waals and electrostatic forces were subtracted from each QM force calculation prior to force-matching so that only bonded terms (i.e. bonds, angles, dihedrals, etc.) need fit. Force matching was then conducted via the ForceSolve [27] program, which employs Bayesian formalism that identifies the parameters that minimize the negative log-likelihood function based on the observed QM forces. Consequently, three sets of parameters were generated for host–guest systems: (1) MP2/6-31G(d) force-matched parameters for the guests and B3LYP/6-31G(d) force matched parameters for the host (FM-MP2). (2) CGenFF parameters obtained via ParamChem for the guest and S6 parameters for the host (C36-S6) and 3) PM6 force matched parameters for the guest and S6 parameters for the host (FM-PM6).

### Replica exchange umbrella sampling (REUS)

In the REUS simulation, the Hamiltonian for the $i^{th}$ replica with umbrella potential $V_{m} \left( {q^{i} } \right)$ can be written as:

3 $$H_{m} \left( {q^{i} ,p^{i} } \right) = K\left( {p^{i} } \right) + E_{{\lambda_{m} }} \left( {q^{i} } \right)$$

where $q^{i}$ and $p^{i}$ are the generalized coordinates and momenta of the system, respectively. The replica biased potential energy $E_{{\lambda_{m} }}$ can be written as:

4 $$E_{{\lambda_{m} }} \left( {q^{i} } \right) = E_{0} \left( {q^{i} } \right) + V_{m} \left( {q^{i} } \right)$$

where $E_{0}$ is the original potential and $V_{m}$ is the umbrella potential. A harmonic potential is used for $V_{m}$ using a reaction coordinate $\zeta$.

5 $$V_{m} \left( q \right) = k_{m} \left( {\zeta \left( q \right) - d_{m} } \right)^{2} m = 1, \ldots , M$$

where $d_{m}$ is the center of umbrella potential and $k_{m}$ is the strength of restraining potential. Since the replicas are noninteracting, the weight factor of state X in the generalized ensemble can be calculated by multiplying the Boltzmann factor of each replica

6 $$W_{{MREM}} \left( X \right) = \mathop \Pi \limits_{{i = 1}}^{M} \exp \left\{ { - \beta H_{mi} \left( {q^{i} ,p^{i} } \right)} \right\} = \exp \left\{ { - \sum\limits_{{i = 1}}^{M} {\beta H_{mi} \left( {q^{i} ,p^{i} } \right)} } \right\}$$

$m_{i}$ is the permutation function connecting replica $i$ with parameter label $m$.

The following defines the criterion for replica exchange from state $X$ to state $X^{\prime}\left( w \right)$ the umbrella potentials $V_{m}$ and $V_{m + 1}$ are exchanged between replicas $i$ and $j$ in state $X^{\prime}:$

7 $$w\left( {X \to X^{\prime}} \right) = w\left( {x_{m}^{i} , x_{m + 1}^{j} } \right) = \left\{ {\begin{array}{ll} {1 for \Delta \le 0} \\ {\exp \left( { - \Delta} \right)\,{\text{for}}\,\Delta > 0} \\ \end{array} } \right.$$

where

8 $${\Delta } = \beta \left( {V_{m} \left( {q^{j} } \right) - V_{m} \left( {q^{i} } \right) - V_{m + 1} \left( {q^{j} } \right) + V_{m + 1} \left( {q^{i} } \right)} \right)$$

The new umbrella potentials $V_{m + 1} (q^{i}$) and $V_{m} \left( {q^{j} } \right)$ are evaluated with the exchanged coordinates. The Weighted Histogram Analysis Method (WHAM) is used to obtain the canonical distributions [9, 11]. WHAM equations were used to reweight the REUS results and obtain the free energy of pulling the ligand into the binding region$, {\Delta G}^{REUS}$. In the REUS simulations, a cylindrical restraint was used to keep the guest molecules in a cylinder defined by the carbonyl oxygen groups on the two sides of cylindrical host. This restraint prevents unwanted interactions of the guest with the side of host molecule and facilitates the convergence of the free energy profile. This cylindrical restraint was chosen as a radial distance of $7.5 {\AA}$ from the principal axis of cylindrical host with a force constant of 5 kcal/mol Å^2^.

**Fig. 2:**
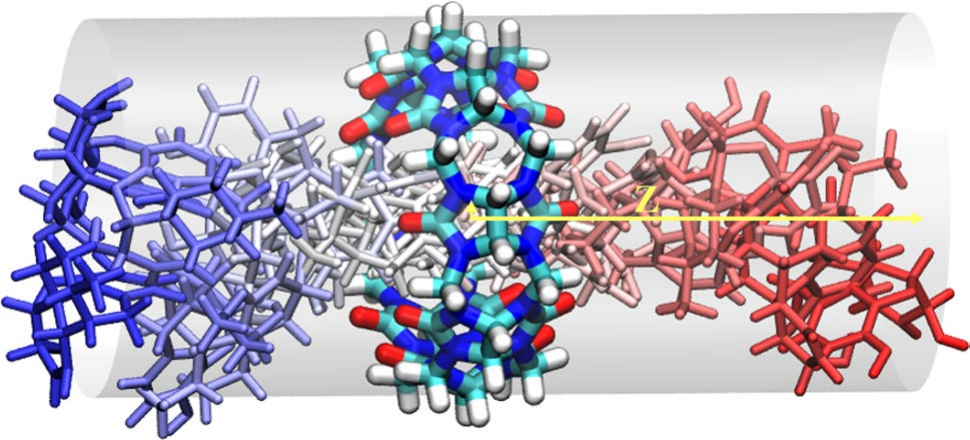
Schematic of REUS simulation with cylindrical restraint (shown in grey) and replicas along reaction coordinate (in yellow). The blue molecule on the left demonstrates a guest-ligand being pulled from the left side of the host (middle) to the right side (red) along the reaction coordinate inside a cylindrical restraint potential (grey)

The reaction coordinate for all systems was defined as the distance between the center of mass of the guest molecule with center of mass of carbonyl oxygens at the left side of the cylindrical host (Fig. 2). The reaction coordinate was divided into 32 umbrellas from -13 to 14 ${\AA}$. We assigned more umbrellas for the reaction coordinates between -3 to 3 rather than an equidistant reaction coordinate to sample the bound poses more than the unbound ones. In REUS, exchange attempts were made every $2{\text{ ps}}$. Simulations for REUS were run for 10–20 ns for each replica. A summary of all REUS simulations can be found in Table S1.

### Correction terms to free energy

Two different correction terms are needed to complete the thermodynamic cycle and compute the free energy of binding. The first correction term is the free energy associated with releasing the restraints on the guest molecule in the unbound state. This is also called the volume correction term, and was assumed as ${\Delta }G^{{{\text{rest}} - {\text{off}}}} = - k_{B} Tln\left( {V_{0} /V_{eff} } \right)$ [33, 38–42]. In this equation $k_{B}$ is the Boltzmann constant,T is the temperature in Kelvin and $V_{0}$ is the standard state volume of an ideal gas at 298 K which is 1649.76 ${\AA}^{3} .$ $V_{{{\text{eff}}}}$ is the effective volume of the guest molecule at the unbound state which is estimated using equation:

9 $$V_{{{\text{eff}}}} = \pi \left( {r_{max}^{2} - r_{min}^{2} } \right)h_{max}$$

where $r_{max}$ and $r_{min}$ are the maximum and minimum radial distance of center of mass of the guest with respect to the principal axis of the cylindrical restraint around host at the first replica (unbound state) and $h_{max}$ is the maximum distance that the center of mass of guest molecule moves along the principal axis of the cylindrical restraint which is also calculated for the first replica.

Another correction to free energy is the energetic cost of turning on the restraint between the host and the ligand at the bound pose, ${\Delta }G^{{{\text{rest}} - {\text{on}}}}$. This was calculated with thermodynamic integration (TI). To compute this, we selected 5 different bound poses from the state with the highest binding affinity. 10 different lambda values 1, 0.95, 0.9, 0.85, 0.8, 0.7, 0.5, 0.3, 0.1, 0 were chosen to perform the TI calculations. For each lambda state, we performed equilibration and Molecular Dynamics (MD) simulation at NVT ensemble for 100 and 200 ${\text{ps}}$ respectively. The correction term is then calculated using the following thermodynamic integration formula and averaged over the 5 chosen binding poses. The cylindrical restraint does not affect the TI calculations since the center of mass of the guest is always inside the cylindrical potential around the host in all bound poses by definition.

10 $${\Delta }G_{{{\text{rest}} - {\text{on}}}}^{{{\text{TI}}}} = \mathop \smallint \limits_{0}^{1} < \frac{\partial V\left( \lambda \right)}{{\partial \lambda }} >_{\lambda } d\lambda$$

The final binding free energy ${\Delta }G^{{{\text{bind}}}}$ is then calculated as:

11 $${\Delta }G^{{{\text{bind}}}} = - {\Delta }G^{{{\text{REUS}}}} - {\Delta }G^{{{\text{rest}} - {\text{off}}}} - {\Delta }G^{{{\text{rest}} - {\text{on}}}}$$

#### Multiple protonation states for G5 guest

**Fig. 3:**
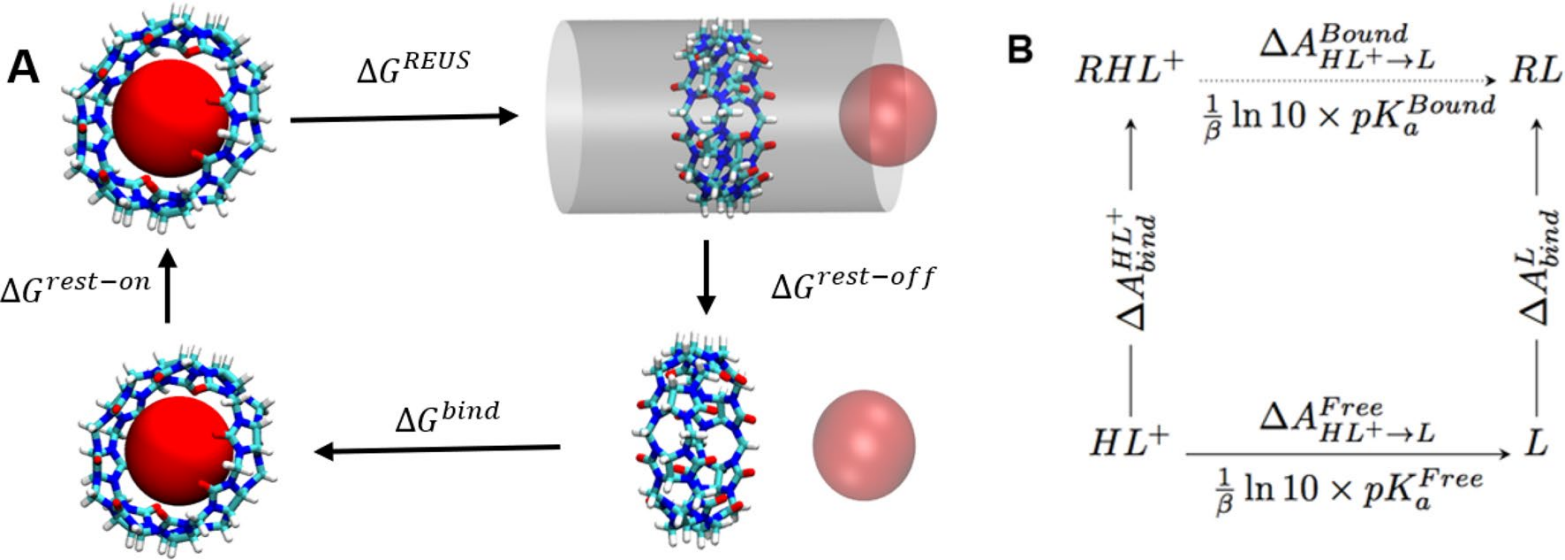
**a** Thermodynamic cycle for the REUS simulations. **b** Thermodynamic cycle for pH correction

The protonation state of G5 (ketamine) is unclear since the ${\text{pK}}_{{\text{a}}}$ is 7.5 which is close to the experimental ${\text{pH}}$ of 7.4 [43]. Due to this uncertainty for protonation state of G5, we have produced force field parameters for both protonated and unprotonated species and performed REUS simulation for both protonation states and both stereoisomers of this molecule. The final free energy at pH of 7.4 is then calculated based on a thermodynamic cycle represented in Fig. 3b. The free energy for each protonation state is computed as the average of R and S stereoisomer binding free energies. The 4 different states of G5 are denoted as G5NR (neutral and R isomer), G5NS (neutral and S isomer, G5PR (protonated and R isomer) and G5PS (protonated and S isomer). In the thermodynamic cycle of Fig. 3b, $L$ stands for unprotonated ligand, $HL^{ + }$ is the protonated ligand and R is the receptor (CB8). ${\Delta }A_{bind}^{L}$ and ${\Delta }A_{bind}^{{HL^{ + } }}$ are the binding free energies for unprotonated and protonated ligands respectively. The deprotonation free energies are computed from the $pK_{a}$ of the ligand in the free state and in the bound state. Finally, the binding free energy of the partially protonated ligand in the bound state can be derived. The equations for the binding free energy at pH 7.4 are:

12 $$pK_{a}^{Bound} = pK_{a}^{Free} + \frac{\beta }{log10}{{\Delta \Delta }}A_{bind}^{{HL^{ + } \to L}}$$

13 $${\Delta }G_{bind}^{pH = 7.4} = {\Delta }G_{bind}^{{HL^{ + } }} - \frac{1}{\beta }\log \left[ {\frac{{1 + 10^{{7.4 - pK_{a}^{Bound} }} }}{{1 + 10^{{7.4 - pK_{a}^{Free} }} }}} \right]$$

### Molecular dynamics simulations

All host–guest systems were solvated in a TIP3P water box with dimension of ~ $68 \times 68 \times 68 {\AA}^{3}$. Sodium and Chloride ions were added to reach the experimental buffer condition (ionic strength) 20 mM sodium phosphate buffer at pH 7.4 and temperature 298.15 K (as well as any extra needed to maintain charge neutrality of the system). Solvated systems were first minimized using NAMD [44]. A 1 ns equilibration was performed using Langevin thermostat with a friction coefficient of 1 ${\text{ps}}^{ - 1}$ and a time step of 1 fs and NVT ensemble. A TIP3P water model was used for all simulations [45]. In the next step a steered MD (SMD) simulation was performed by applying an external force with 5 kcal/mol Å^2^ to the heavy atoms of the guest molecule along the reaction coordinate to transfer from one side of the cylindrical host ( value of reaction coordinate -13) to the other side ( reaction coordinate of + 14). This bidirectional approach was possible due to symmetry of the host, large opening in the center and small guest molecules. A time step of 2 fs was used with the SHAKE algorithm for bonds involving hydrogen atoms [46]. A force-switching function [47] was used to truncate van der Waals interactions smoothly at 10–12 ${\AA}$ and the long-range electrostatic interactions were calculated using the particle mesh Ewald (PME) method [48]. The temperature was maintained at 298.15 K using a Langevin thermostat with a friction constant of 1 ${\text{ps}}^{ - 1}$ and the pressure was maintained at 1 bar using a Nose–Hoover Langevin piston [49] method with a period of 50 fs and piston decay of 25 fs.

## Results and discussion

Table 1 shows the computed free energies of pulling (REUS), as well as correction terms for the FM-MP2 force-matched parameter set. The average binding free energies computed with three different parameter sets are shown in Table 2 and compared with the experimental values. The reaction coordinate in all simulations is the distance between the center of mass of the heavy atoms of the guest molecule and the carbonyl oxygen atoms of the left side of cylindrical host projected along the principal z axis of the cylindrical restraint around the host. A depiction of the restraint and the reaction coordinate is shown in Fig. 2. A bidirectional reaction coordinate was adopted for all systems where replicas are initiated in both sides of the cylindrical host except for G2 system in FM-MP2 force-matched parameter set. A summary of all the simulation parameters is in table S1. An exchange rate of 20–30% was observed for all systems.

**Table 1:** Binding free energy components for FM-MP2 force-matched parameter set in ${\text{kcal}}/{\text{mol}}$. Binding free energies for the isomeric states of G5 are averaged and shown in Avg binding energy column. – in this column means the value is the same as the binding free energy in the previous column

Guest	Volume Correction ${{\varvec{\Delta}}}{\varvec{G}}^{{{\varvec{rest}} - {\varvec{off}}}}$ [kcal/mol]	Restraint on ${{\varvec{\Delta}}}{\varvec{G}}^{{{\varvec{rest}} - {\varvec{on}}}}$ [kcal/mol]	REUS PMF ${{\varvec{\Delta}}}{\varvec{G}}^{{{\varvec{REUS}}}}$ [kcal/mol]	Binding free energy ${{\varvec{\Delta}}}{\varvec{G}}^{{{\varvec{bind}}}}$ [kcal/mol]	Avg binding free energy [kcal/mol]
G1	$- 0.74 \pm 0.11$	$0.30 \pm 0.08$	$- 12.30 \pm 0.47$	$- 12.75 \pm 0.67$	$-$
G2	$- 0.42 \pm 0.31$	$0.13 \pm 0.01$	$- 3.53 \pm 0.44$	$- 3.82 \pm 0.75$	$-$
G3	$- 0.64 \pm 0.11$	$0.17 \pm 0.01$	$- 14.86 \pm 0.76$	$- 15.33 \pm 0.87$	$-$
G4	$- 0.70 \pm 0.34$	$0.16 \pm 0.02$	$- 17.07 \pm 0.62$	$- 17.62 \pm 0.98$	$-$
G5NR	$- 0.68 \pm 0.11$	$0.48 \pm 0.29$	$- 4.02 \pm 0.47$	$- 4.21 \pm 0.87$	$- 4.10 \pm 1.05$
G5NS	$- 0.67 \pm 0.11$	$0.68 \pm 0.43$	$- 3.99 \pm 0.69$	$- 3.98 \pm 1.23$	
G5PR	$- 0.69 \pm 0.11$	$0.39 \pm 0.14$	$- 13.00 \pm 0.49$	$- 13.30 \pm 0.74$	$- 13.26 \pm 0.76$
G5PS	$- 0.69 \pm 0.11$	$0.18 \pm 0.06$	$- 12.72 \pm 0.59$	$- 13.22 \pm 0.77$	
G6	$- 0.69 \pm 0.11$	$0.18 \pm 0.01$	$- 16.42 \pm 0.54$	$- 16.93 \pm 0.66$	$-$
**G7**	$- 0.62 \pm 0.11$	$0.23 \pm 0.18$	$- 12.06 \pm 0.64$	$- 12.44 \pm 0.92$	$-$

**Table 2:** Average binding free energies ${\Delta }G^{{{\text{bind}}}}$ [${\text{kcal}}/{\text{mol}}]$ for the three parameter sets used here as compared with the experimental binding free energies. G5N is the unprotonated and G5P is the protonated G5. G5* is the binding free energy after applying the $pK_{a}$ corrections

Guest	FM-MP2 [kcal/mol]	C36-S6 [kcal/mol]	FM-PM6 [kcal/mol]	Experimental [kcal/mol]
G1	$- 12.75 \pm 0.67$	$- 10.25 \pm 0.69$	$- 9.90 \pm 0.90$	$- 7.05 \pm 0.04$
G2	$- 3.82 \pm 0.75$	$- 8.38 \pm 0.75$	$- 8.38 \pm 0.65$	$- 9.93 \pm 0.03$
G3	$- 15.33 \pm 0.87$	$- 15.95 \pm 0.63$	$- 11.75 \pm 0.88$	$- 11.63 \pm 0.03$
G4	$- 17.62 \pm 0.98$	$- 14.96 \pm 1.00$	$- 11.93 \pm 0.64$	$- 11.22 \pm 0.04$
G5N	$- 4.10 \pm 1.05$	$- 11.32 \pm 1.03$	$- 10.41 \pm 1.05$	$-$
G5P	$- 13.26 \pm 0.76$	$- 8.73 \pm 0.78$	$- 11.70 \pm 1.06$	$-$
G5*	$- 12.91 \pm 0.91$	$- 10.90 \pm 0.91$	$- 11.40 \pm 1.05$	$- 12.32 \pm 0.04$
G6	$- 16.93 \pm 0.66$	$- 12.56 \pm 0.63$	$- 14.23 \pm 0.61$	$- 14.07 \pm 0.06$
G7	$- 12.44 \pm 0.92$	$- 8.25 \pm 1.03$	$- 10.08 \pm 0.84$	$- 7.92 \pm 0.04$

We adopted the same bidirectional approach to compute REUS free energy of pulling for all systems. However, for G2 with FM-MP2, the two-sided free energy didn’t converge symmetrically, and a unidirectional approach was used. This was likely due to the larger cylindrical restraint radius used for this system which caused G2 to interact mostly with the sides of the host and cause divergence of free energy. Average binding free energies for all three sets of parameters are shown and compared with the experimental values in Table 2.

Tables S2 and S3 show the free energies of binding for FM-PM6 and C36-S6 force-matched parameter sets. The reaction coordinate used for these two systems were the same with a cylindrical restraint 7.5 ${\AA}$ around the center of mass of the cylindrical host and 32 replicas along the reaction coordinate. The simulation time and parameters are shown in table S1. The statistical measurements including RMSE, Kendall’s tau ($\tau$) rank correlation coefficient which measures the relative ranking between the computed and experimental free energies (values closer to 1 implies nearly identical hierarchal ordering between sets) and correlation coefficient ($R^{2}$) as well as Pearson’s correlation $r$ were measured for the three force-matched parameter sets and shown in Table 3.

**Table 3:** Statistical measurements between the calculated free energies (bidirectional) and the experimental values. MAE is the mean absolute error and ME is the mean error

Parameter set	${\varvec{R}}^{2}$	${\varvec{r}}$	${\varvec{RMSE}}$	${\varvec{\tau}}$	MAE	ME
FM-MP2	$0.16$	$0.40$	$4.68$	$0.33$	2.07	2.52
C36-S6	$0.31$	$0.55$	$2.65$	$0.43$	1.51	1.009
FM-PM6	$0.50$	$0.71$	$1.72$	$0.52$	1.14	0.61

In the FM-MP2 set of free energies, correlation coefficient was low (0.16), RMSE was 4.68 ${\text{kcal}}/{\text{mol}}$ and $\tau$ was 0.33. In this set of simulations, all binding free energies are overestimated except for G2 which is due to inadequate sampling in the unidirectional free energy for this molecule. While the results from FM-MP2 set were unsatisfactory, having a low statistical significance (Pearson’s correlation was 0.40), the binding free energies for this parameter set correlate more closely to the results obtained via the double decoupling approach used by Hudson et. al. in the SAMPL8 challenge (in a forthcoming manuscript detailing the effort). For the simulations utilizing C36-S6 parameters, overall, the predictions of the binding free energies improved, with an increase in the correlation coefficient by 0.15 units compared to FM-MP2 set. The RMSE and $\tau$ values determined for C36-S6 set, $2.65 {\text{kcal}}/{\text{mol}}$ and 0.43, which were better than the results from FM-MP2. Changing force field parameters to FM-PM6 resulted in even more accurate binding free energies with RMSE of $1.72 {\text{kcal}}/{\text{mol}}$ and $\tau$ of 0.52.

For both C36-S6 and the FM-PM6 parameter sets, the bidirectional free energy computations converged for G2. The free energies for both parameters set for this guest were very similar ( $- 8.4 {\text{kcal}}/{\text{mol}}$), which shows that the bidirectional free energy profile is better suited for this large molecule. In FM-MP2 we increased the size of the cylindrical restraint for G2 which resulted in interaction of G2 with the sides of CB8 and the bidirectional free energy didn’t converge. We also tested the unidirectional free energy REUS for G1, G3 and G4 with PM6-S6 parameter set. Table S4, compares the binding free energies between unidirectional and bidirectional REUS methods. The free energies for these two approaches are highly similar which means the unidirectional approach is better suited due to the lower computational cost associated with a smaller number of replicas (as well as the inherit use for non-symmetric hosts).

**Fig. 4:**
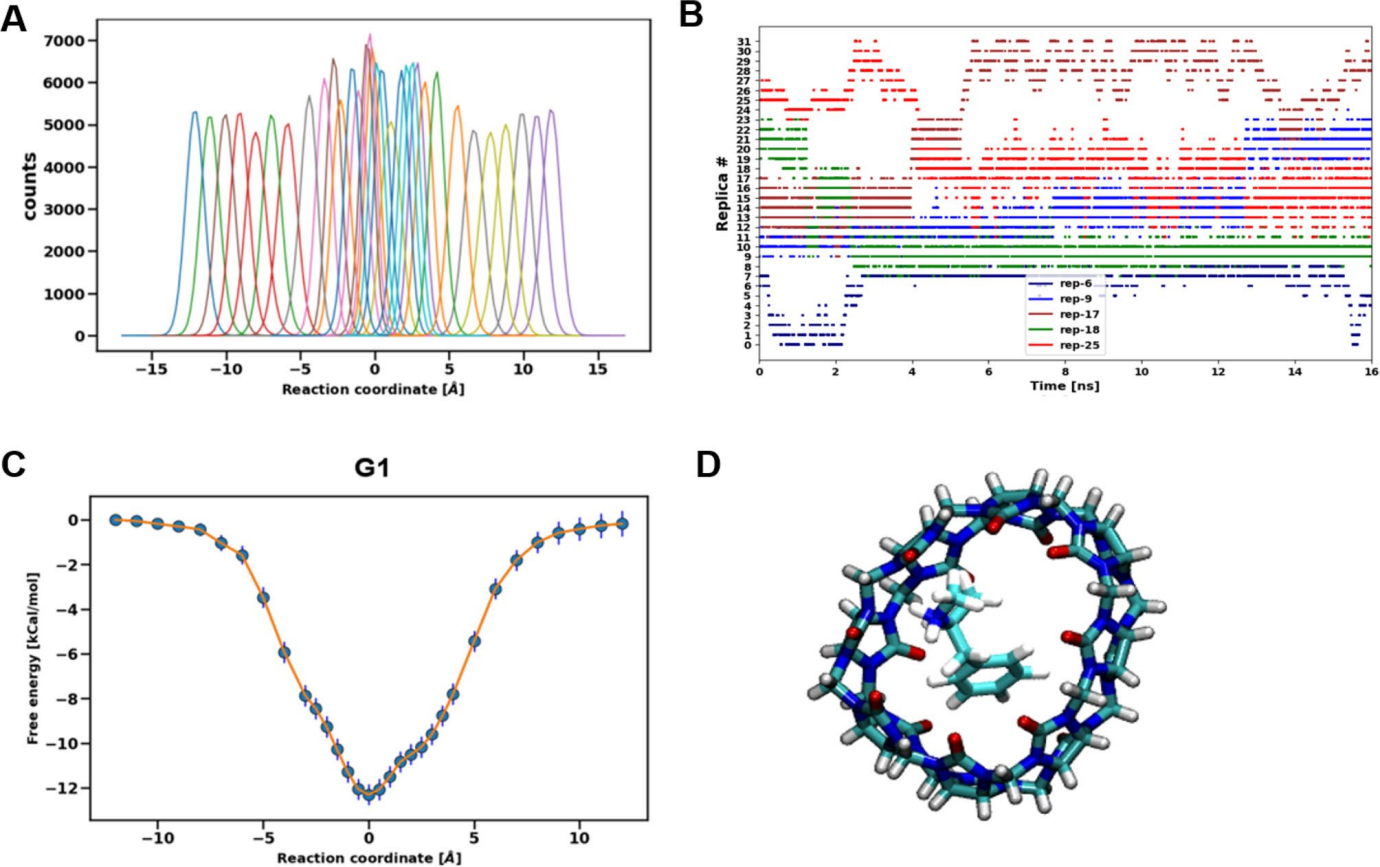
**a** Histograms of reaction coordinate for G1 in FM-MP2 set showing high amount of overlap. **b** Time series of five selected replicas [6, 9, 17, 18, 25] in REUS for G1 showing high exchange **c** free energy profile along the reaction coordinate in the bidirectional approach for G1 **d** bound pose for G1 in FM-MP2 parameter set

Figure 4a shows the distribution of reaction coordinates for replicas in G1-host system with FM-MP2 parameter set (a representative system), where each color corresponds to an umbrella potential after sorting the replicas. Sufficient overlap in the distribution of umbrella centers is observed so we can successfully use reweighting methods to calculate the PMFs. The time series of replica-exchange for a few selected replicas as shown in Fig. 4b, reflect the center of umbrella potentials in the reaction coordinate. This shows the replica exchange simulations were appropriately performed and exchange happens from the unbound state where the guest has conformational freedom to the bound state where the guest is restricted. The replicas in the bound region are exchanging between both sides of the cylindrical host which leads to a symmetric PMF in Fig. 4c. REUS simulation performs random walks not only in the reaction coordinate space but also in the conformational space of host and guest systems as multiple conformations in the bound state are observed. An average exchange rate of 25–30% was observed for all replicas in all systems which shows the parameters chosen for REUS were appropriate to get sufficient exchange rate. Figure 4c shows the PMF of G1 in FM-MP2 simulations. The free energy profile is symmetric with respect to the bound pose at replica 0. The bound pose of G1-host system at replica 0 is shown in Fig. 4d.

**Fig. 5:**
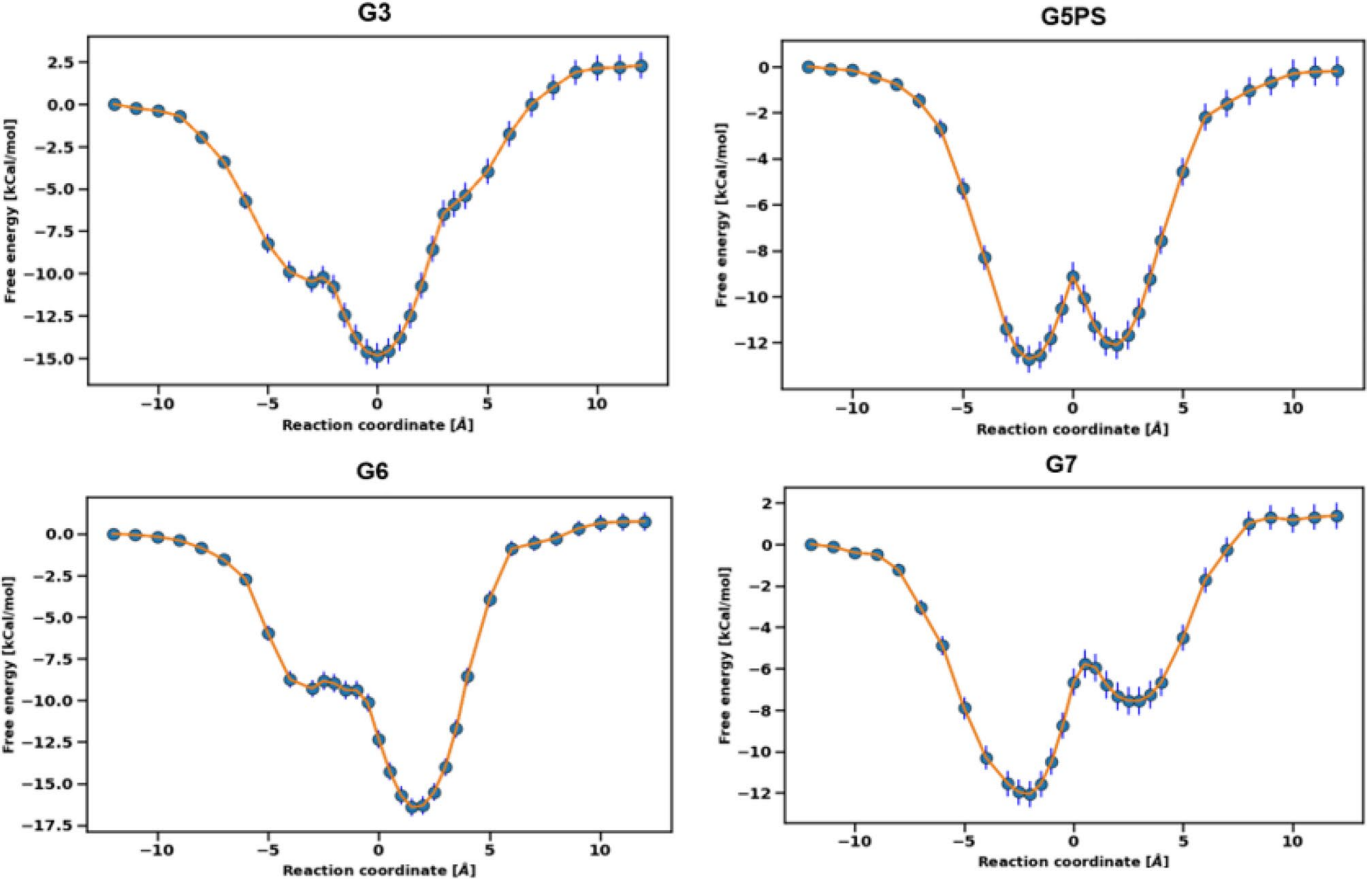
free energy profiles for G3, G5PS, G6 and G7 guest molecules for FM-MP2 parameter set

Symmetry of the free energy profiles in the bidirectional approach with respect to the bound pose can be achieved for the small guest molecules such as G1 (Fig. 4c). However, for larger guest molecules such G2, G6 and G7 the profile is mostly asymmetric. This is mainly due to insufficient sampling for these larger systems as there are more degrees of freedom to be explored by the REUS simulation. The pulling of the guest molecule through the cylindrical host to the other side is a rare event which causes an asymmetric profile and running longer REUS simulation may help alleviate this issue. Moreover, the exchange between the two sides of the host is compromised for larger guest molecules. In Fig. 5 we show the PMF for a few systems in the FM-MP2 parameter set. This is a representative example and other parameter sets show the same behavior in the free energy profiles.

REUS pulling simulations were also performed by using a unidirectional reaction coordinate approach for PM6-S6 parameter set and the pulling free energies were compared with the bidirectional approach for a few molecules [G1,G3,G4]. The free energies obtained from one-sided PMF were similar to the two-sided approach for G1, G3 and G4 molecules with less than $0.5 {\text{kcal}}/{\text{mol}}$ difference. The PMF for G3 in the FM-PM6 bidirectional approach was asymmetric and the similarity between the unidirectional and bidirectional free energies show that this asymmetry does not affect the value of the binding free energy. Thus, to obtain a converged PMF with a smaller number of replicas a unidirectional free energy approach suffices for better convergence and less computational cost.

**Fig. 6:**
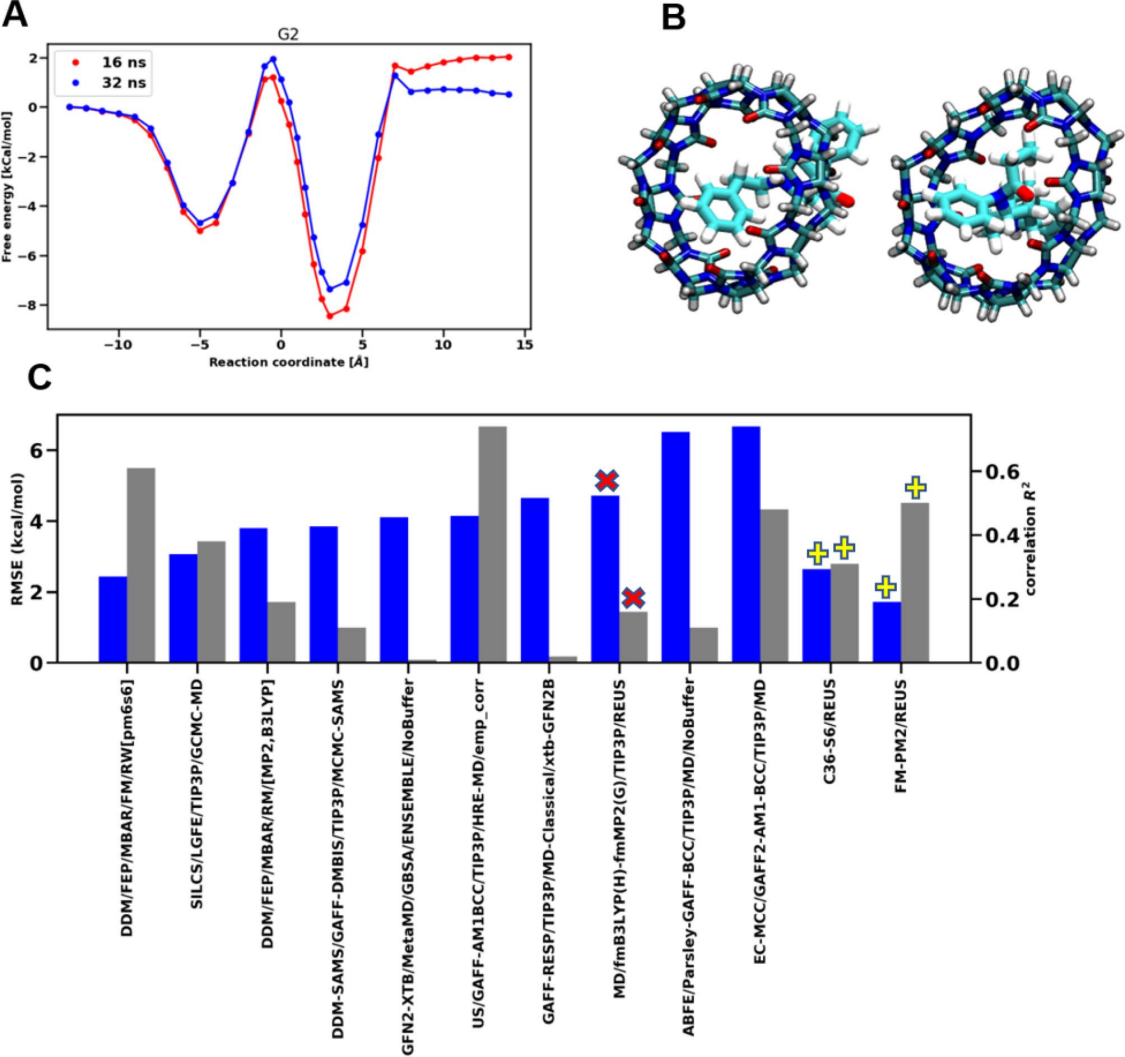
**a** PMF of G2 binding to host in PM6-S6 parameter set after 16 and 32 ns. **b** bound poses for two different minima in the PMF of G2 after 32 ns. **c** RMSE and $R^{2}$ for all ranked submissions in SAMPL8 challenge. The submission from this paper is shown with a $\times$ sign on top of bar and the results with C36-S6 and FM-MP2 are shown with $+$ sign. Blue bars show the RMSE and gray bars show the $R^{2}$

Testing how well the free energy profile of G2 with FM-PM6 parameters as a representative of larger guest molecules, we extended the simulation with a bidirectional free energy approach and compared the free energy profiles obtained after 16 and 32 ns in Fig. 6a. It is seen that after 32 ns the profile is getting more symmetric and the right side of the profile is flattening out. This is due to more exchange between right and left side of the host which lead to a more symmetric profile. Furthermore, the two minima free energies are closer after 32 ns of simulation. Figure 6b shows the binding poses of G2 from the left side of PMF (left) and right side of PMF (right). One can see that for both cases, G2 binds differently with the Host molecule.

Table 3 compares the results of the three different parameter sets used for the REUS calculations and the statistics with respect to the experimental binding affinities. Figure 6c compares the ranked submissions in SAMPL8 in terms of RMSE and $R^{2}$. Although the results from FM-MP2 parameter set showed poor statistical accuracy with respect to the experimental binding affinities, these results highly similar to those obtained with double decoupling approach by Hudson et. al in SAMPL8 using the same parameter set. To show the effectiveness of REUS to accurately calculate binding free energies, we next used two other parameter sets (C36-S6 and PM6-S6) that already have shown better correlation with experimental binding affinities from the double decoupling method (detailed in a forth-coming paper regarding a seperate SAMPL8 submission). The RMSE from C36-S6 and FM-PM6 parameter set were $2.65 {\text{kcal}}/{\text{mol}}$ and $1.72 {\text{kcal}}/{\text{mol}}$ respectively. This indicates that the ParamChem-generated CGenFF parameters are a good starting point for calculating binding affinities with a proper sampling approach such as REUS. The results from FM-PM6 parameter set have better RMSE than all the ranked submissions in challenge which indicates that using appropriate force-matched parameters along with REUS can yield a high correlation with experimental binding affinities. Furthermore, we have showed that a unidirectional approach yields similar binding affinities as to a bidirectional approach but with lower number of replicas and consequently a lower computational cost.

## Conclusion

In this paper we have used a REUS approach for binding free energy calculations of host–guest systems in SAMPL8 blind challenge. Multiple force matched parameter sets were applied to the host–guest systems. In the first attempt, the guest molecules parameters were force-matched to FM-MP2. We later used another set of parameters FM-PM6 or with ParamChem based C36-S6 parameters. Although the first attempt with FM-MP2 resulted in overestimation of free energies compared to experimental values, following attempts with C36-S6 and FM-PM6 led to better results with RMSE values of $2.65$ and $1.72 {\text{kcal}}/{\text{mol }}$ respectively. Furthermore, we showed that convergence of free energy profile can be obtained with a unidirectional approach rather than a bidirectional one, where the guest molecule is pulled through the lumen of CB8. The unidirectional approach requires smaller number of replicas and led to similar binding free energies for host–guest systems. Larger guest molecules require more sampling for convergence of free energy profiles. This could be achieved by adding the time of REUS simulation. We are, therefore, optimistic that REUS simulations can provide highly accurate results with the proper description of energetics of the system.

## Supplementary Information

Below is the link to the electronic supplementary material.

Supplementary file1 (DOCX 25 kb)
