# Supplementary material for: A replica exchange umbrella sampling (REUS) approach to predict host–guest binding free energies in SAMPL8 challenge
Source: J Comput Aided Mol Des. 2021 May 3;35(5):667–77. doi: 10.1007/s10822-021-00385-7 (PMC8131287; doi:10.1007/s10822-021-00385-7)
Supplement: Supplementary file 1 — Supplementary file1 (DOCX 25 kb) [file 10822_2021_385_MOESM1_ESM.docx]

Supplementary information for :

**A replica exchange umbrella sampling (REUS) approach to predict host-guest binding free energies in SAMPL8 challenge**

Mahdi Ghorbani^1,2*^, Philip S. Hudson^1^, Félix Aviat^1^, Rubén Meana Pañeda^1^, Michael R. Jones^1^, Jeffery B. Klauda^2^, Bernard R. Brooks^1^

^1^ Laboratory of Computational Biology, National Heart, Lung and Blood Institute, National Institutes of H

ealth, Bethesda, MD 20892, USA

^2^ University of Maryland, Department of Chemical and Biomolecular Engineering, College Park 20740, USA

*corresponding author

**Table S1.** Simulation times for SAMPL8 host-guest systems per parameter set.

**Table S2.** Contributions to the binding free energy for the C36-S6 parameter set.

**Table S3.** Contributions to the binding free energy for the PM6-S6 parameter set.

**Table S4.** Comparison of one-sided and two-sided REUS free energies for G1, G3 and G4 using the FM-PM6 parameter set.

**Table S1.** Simulation times for SAMPL8 host-guest systems per parameter set.

| Host-Guest System | FM-MP2 | C36-S6 | FM-PM6 |
| --- | --- | --- | --- |
| G1-CB8 | 16 ns | 20 ns | 10 ns |
| G2-CB8 | 20 ns | 20 ns | 32 ns |
| G3-CB8 | 18 ns | 20 ns | 10 ns |
| G4-CB8 | 16 ns | 20 ns | 10 ns |
| G5NR-CB8 | 20 ns | 20 ns | 10 ns |
| G5NS-CB8 | 20 ns | 20 ns | 10 ns |
| G5PR-CB8 | 20 ns | 20 ns | 10 ns |
| G5PS-CB8 | 20 ns | 20 ns | 10 ns |
| G6-CB8 | 20 ns | 20 ns | 10 ns |
| G7-CB8 | 20 ns | 20 ns | 10 ns |

**Table S2.** Contributions to the binding free energy for the C36-S6 parameter set.

| Guest | Volume Correction $\boldsymbol{\Delta}\boldsymbol{G}^{\boldsymbol{rest-off}}$ | Restraint On $\boldsymbol{\Delta}\boldsymbol{G}^{\boldsymbol{rest-on}}$ | REUS PMF $\boldsymbol{\Delta}\boldsymbol{G}^{\boldsymbol{REUS}}$ | Binding Free Energy $\boldsymbol{\Delta}\boldsymbol{G}^{\boldsymbol{bind}}$ | Avg Binding Free Energy |
| --- | --- | --- | --- | --- | --- |
| **G1** | $-0.79\pm0.11$ | $0.62\pm0.04$ | $-10.08\pm0.54$ | $-10.25\pm0.69$ | $-$ |
| **G2** | $-0.88\pm0.12$ | $0.17\pm0.09$ | $-7.66\pm0.54$ | $-8.38\pm0.75$ | $-$ |
| **G3** | $-0.75\pm0.11$ | $0.17\pm0.01$ | $-15.37\pm0.51$ | $-15.95\pm0.63$ | $-$ |
| **G4** | $-0.76\pm0.11$ | $0.25\pm0.10$ | $-14.46\pm0.79$ | $-14.96\pm1.00$ | $-$ |
| **G5NR** | $-0.69\pm0.11$ | $0.53\pm0.20$ | $-13.42\pm0.88$ | $-13.58\pm1.19$ | $-6.82\pm1.03$ |
| **G5NS** | $-0.70\pm0.11$ | $0.34\pm0.09$ | $-8.69\pm0.66$ | $-9.05\pm0.87$ |  |
| **G5PR** | $-0.68\pm0.11$ | $0.33\pm0.06$ | $-7.98\pm0.55$ | $-8.33\pm0.72$ | $8.73\pm0.78$ |
| **G5PS** | $-0.69\pm0.11$ | $0.30\pm0.02$ | $-8.73\pm0.72$ | $-9.12\pm0.85$ |  |
| **G6** | $-0.75\pm0.12$ | $0.14\pm0.02$ | $-11.95\pm0.49$ | $-12.56\pm0.63$ | $-$ |
| **G7** | $-0.73\pm0.11$ | $0.49\pm0.26$ | $-8.02\pm0.65$ | $-8.25\pm1.03$ | $-$ |

**Table S3.** Contributions to the binding free energy for the PM6-S6 parameter set.

| Guest | Volume Correction $\boldsymbol{\Delta}\boldsymbol{G}^{\boldsymbol{rest-off}}$ | Restraint On $\boldsymbol{\Delta}\boldsymbol{G}^{\boldsymbol{rest}\boldsymbol{-}\boldsymbol{on}}$ | REUS PMF $\boldsymbol{\Delta}\boldsymbol{G}^{\boldsymbol{REUS}}$ | Binding Free Energy $\boldsymbol{\Delta}\boldsymbol{G}^{\boldsymbol{bind}}$ | Avg Binding Free Energy |
| --- | --- | --- | --- | --- | --- |
| **G1** | $-0.18\pm0.16$ | $0.20\pm0.02$ | $-9.92\pm0.72$ | $-9.90\pm0.90$ | $-$ |
| **G2** | $-0.27\pm0.17$ | $0.34\pm0.15$ | $-8.44\pm0.33$ | $-8.38\pm0.65$ | $-$ |
| **G3** | $-0.19\pm0.17$ | $0.16\pm0.02$ | $-11.71\pm0.70$ | $-11.75\pm0.88$ | $-$ |
| **G4** | $-0.66\pm0.09$ | $0.14\pm0.01$ | $-11.41\pm0.54$ | $-11.93\pm0.64$ | $-$ |
| **G5NR** | $-1.06\pm0.16$ | $0.48\pm0.07$ | $-11.06\pm0.83$ | $-11.64\pm1.06$ | $-10.41\pm1.05$ |
| **G5NS** | $-1.06\pm0.16$ | $0.69\pm0.18$ | $-8.80\pm0.69$ | $-9.17\pm1.04$ |  |
| **G5PR** | $-1.04\pm0.16$ | $0.20\pm0.04$ | $-10.46\pm0.48$ | $-11.30\pm0.68$ | $-11.70\pm1.06$ |
| **G5PS** | $-1.06\pm0.16$ | $0.52\pm0.36$ | $-11.55\pm0.92$ | $-12.09\pm1.43$ |  |
| **G6** | $-1.05\pm0.16$ | $0.14\pm0.01$ | $-13.32\pm0.45$ | $-14.23\pm0.61$ | $-$ |
| **G7** | $-0.83\pm0.11$ | $0.15\pm0.08$ | $-9.40\pm0.65$ | $-10.08\pm0.84$ | $-$ |

**Table S4.** Comparison of one-sided and two-sided REUS free energies for G1, G3 and G4 using the FM-PM6 parameter set.

| Host Guest System | One-sided $\boldsymbol{\Delta}\boldsymbol{G}^{\boldsymbol{REUS}}$ | Two-sided $\boldsymbol{\Delta}\boldsymbol{G}^{\boldsymbol{REUS}}$ |
| --- | --- | --- |
| G1-CB8 | $-9.87\pm0.73$ | $9.92\pm0.72$ |
| G3-CB8 | $-11.69\pm1.13$ | $11.71\pm0.70$ |
| G4-CB8 | $-11.80\pm1.26$ | $11.41\pm0.54$ |
